# Review of *Cryptosporidium* and *Giardia* in the eastern part of Europe, 2016

**DOI:** 10.2807/1560-7917.ES.2018.23.4.16-00825

**Published:** 2018-01-25

**Authors:** Judit Plutzer, Brian Lassen, Pikka Jokelainen, Olgica Djurković-Djaković, István Kucsera, Elisabeth Dorbek-Kolin, Barbara Šoba, Tamás Sréter, Kálmán Imre, Jasmin Omeragić, Aleksandra Nikolić, Branko Bobić, Tatjana Živičnjak, Snježana Lučinger, Lorena Lazarić Stefanović, Jasmina Kučinar, Jacek Sroka, Gunita Deksne, Dace Keidāne, Martin Kváč, Zuzana Hůzová, Panagiotis Karanis

**Affiliations:** 1 Department of Water Hygiene, National Public Health Institute, Budapest, Hungary; 2 Department of Basic Veterinary Sciences and Population Medicine, Institute of Veterinary Medicine and Animal Science, Estonian University of Life Sciences, Tartu, Estonia; 3 Department of Veterinary Disease Biology, University of Copenhagen, Frederiksberg, Denmark; 4 Faculty of Veterinary Medicine, University of Helsinki, Helsinki, Finland; 5 Department of Bacteria, Parasites & Fungi, Infectious Disease Preparedness, Statens Serum Institut, Copenhagen, Denmark; 6 Centre of Excellence for Food- and Vector-borne Zoonoses, Institute for Medical Research, University of Belgrade, Belgrade, Serbia; 7 Department of Parasitology, National Public Health Institute, Budapest, Hungary; 8 Institute of Microbiology and Immunology, Faculty of Medicine, University of Ljubljana, Ljubljana, Slovenia; 9 National Food Chain Safety Office, Veterinary Diagnostic Directorate, Budapest, Hungary; 10 Banat's University of Agricultural Sciences and Veterinary Medicine ‘King Michael I of Romania’ from Timişoara, Faculty of Veterinary Medicine, Department of Animal Production and Veterinary Public Health, Timişoara, Romania; 11 University of Sarajevo, Veterinary Faculty, Department of Parasitology and Invasive Diseases of Animals, Sarajevo, Bosnia and Herzegovina; 12 Department for Parasitology and Parasitic Diseases with Clinic, Faculty of Veterinary Medicine, University of Zagreb, Zagreb, Croatia; 13 Department of Microbiology, Public Health Institute of Istrian Region, Pula, Croatia; 14 Department of Parasitology, National Veterinary Research Institute, Puławy, Poland; 15 Institute of Food Safety, Animal Health and Environment – ‘BIOR’, Riga, Latvia; 16 Faculty of Veterinary Medicine, Latvia University of Agriculture, Jelgava, Latvia; 17 Institute of Parasitology, Biology Centre of the Czech Academy of Sciences, České Budějovice, Czech Republic; 18 Faculty of Agriculture, University of South Bohemia in České Budějovice, České Budějovice, Czech Republic; 19 Health Institute in Ústí nad Labem, Prague, Czech Republic; 20 State Key Laboratory for Plateau Ecology and Agriculture, Centre for Biomedicine and Infectious Diseases Qinghai University, Xining, China; 21 Medical School, University of Cologne, Cologne, Germany

**Keywords:** One Health, cryptosporidiosis, giardiasis, zoonosis

## Abstract

This paper reviews the current knowledge and understanding of *Cryptosporidium* spp*.* and *Giardia* spp. in humans, animals and the environment in 10 countries in the eastern part of Europe: Bosnia and Herzegovina, Croatia, Czech Republic, Estonia, Hungary, Latvia, Poland, Romania, Serbia and Slovenia. **Methods:** Published scientific papers and conference proceedings from the international and local literature, official national health service reports, national databases and doctoral theses in local languages were reviewed to provide an extensive overview on the epidemiology, diagnostics and research on these pathogens, as well as analyse knowledge gaps and areas for further research. **Results:** *Cryptosporidium* spp. and *Giardia* spp. were found to be common in eastern Europe, but the results from different countries are difficult to compare because of variations in reporting practices and detection methodologies used. **Conclusion:** Upgrading and making the diagnosis/detection procedures more uniform is recommended throughout the region. Public health authorities should actively work towards increasing reporting and standardising reporting practices as these prerequisites for the reported data to be valid and therefore necessary for appropriate control plans.

## Introduction

*Cryptosporidium* spp. and *Giardia* spp. have been ranked as the sixth and 11th most important food-borne parasites globally, respectively [1]. Both parasites are shed in the faeces of infected hosts and can infect new hosts via faecal-contaminated soil, water, feed and food [2]. Several *Cryptosporidium* species are clearly zoonotic, including *C. parvum*, while human giardiasis is caused by two genetically different groups of *G. intestinalis*, referred to as assemblages A and B, which can infect other mammalian hosts and thus have a zoonotic potential [3]. Control of pathogens that can be transmitted among humans, animals and the environment is best achieved with the One Health approach.

Among food-borne diseases, cryptosporidiosis and giardiasis cause a considerable burden at the global level [4], but the burden at regional and national levels is largely unknown [1,5]. Moreover, the current estimates of the burden caused by zoonotic pathogens only include a part of the potential impacts and true costs. In a One Health context, the estimates of disease burden would address that in humans and that in animals, including reduced human and animal health, economic losses, environmental contamination and the impact on biodiversity.

The most common clinical presentation of human cryptosporidiosis is profuse watery diarrhoea with abdominal pain, low-grade fever, nausea, vomiting and weight loss. It is often asymptomatic, mild or self-limiting in immunocompetent individuals and serious, even fatal in immunosuppressed individuals, such as HIV-infected persons [6,7]. A *Cryptosporidium* spp. infection can also be fatal in several mammalian animals and chronic in reptiles [8].

The clinical features of acute giardiasis in humans are similar to cryptosporidiosis, and include severe diarrhoea, abdominal cramps, nausea and weight loss. These symptoms may persist for a few weeks or evolve into a chronic reoccurring disease. The infection may be asymptomatic or a subclinical course [9]. *Giardia* spp. infection in cattle, goats and sheep can cause nutrient malabsorption that can consequently result in a reduction of weight gain. Although mortality due to giardiasis is uncommon, fatal giardiasis has been reported in chinchillas and birds [10].

Microscopic examination of stool specimens remains the cornerstone of diagnostic testing for these parasites, although molecular methods and immunological assays can effectively replace microscopic approaches. Microscopy is cheap, but requires a skilled parasitologist and the diagnostic yield is dependent on proper stool collection. The treatment options for both include antiparasitic drugs and fluid therapy.

According to 2015 data on food-borne and waterborne diseases and zoonoses in the European Centre for Disease Prevention and Control’s (ECDC) Surveillance Atlas of Infectious Diseases, 0.68% (73/10,805; 95% confidence interval (CI): 0.53–0.84) of confirmed cryptosporidiosis cases and 26.71% (4,739/17,740; 95% CI: 26.1–27.4) of confirmed giardiasis cases were reported by 10 countries of the European Union (EU) that are mostly in the eastern part of Europe: Bulgaria, Czech Republic, Estonia, Hungary, Latvia, Lithuania, Poland, Romania, Slovakia and Slovenia [11]. These countries make up 20% of the EU population [12]. Considering that *Cryptosporidium* spp. and *Giardia* spp. are transmitted via similar pathways, and that one fourth of all giardiasis cases notified in the EU were from these 10 countries, the low proportion of cryptosporidiosis cases suggests under-reporting. In general, relatively little is known about the presence of *Cryptosporidium* spp. and *Giardia* spp. in the eastern part of Europe despite their public health relevance. This review aimed to assess the significance of *Cryptosporidium* spp. and *Giardia* spp. infections in humans and animals, as well as their occurrence in the environment based on (locally) available data. While the data are challenging to compare, they provide an overall picture of the situation and main knowledge gaps.

## Methods

For the purpose of this analysis, we considered the following 19 countries to comprise eastern Europe: Estonia, Latvia, Lithuania, Czech Republic, Hungary, Poland, Slovakia, Slovenia, Albania, Bulgaria, Bosnia and Herzegovina, the former Yugoslav Republic of Macedonia, Montenegro, Croatia, Serbia, Belarus, Moldova, Ukraine and Romania (Figure).

**Figure:**
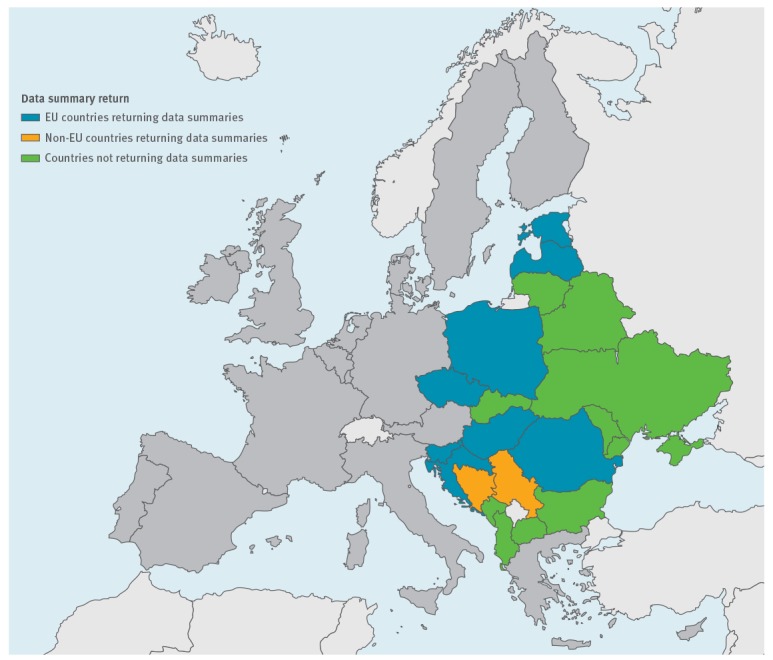
Countries invited to review data on *Cryptosporidium* spp. and *Giardia* spp. from a One Health perspective, 2016

EU: European Union.

Administrative boundaries: EuroGeographics, UN-FAO.

Experts including public health specialists, epidemiologists, parasitologists and other laboratory scientists working on human, animal and environmental samples from the 19 countries were invited in January 2016 to collect and review the data available for their country from a One Health perspective. Experts from 11 of 19 countries responded; those from 10 countries (Bosnia and Herzegovina, Croatia, Czech Republic, Estonia, Hungary, Latvia, Poland, Romania, Serbia, Slovenia) sent summary reviews, while those from the former Yugoslav Republic of Macedonia responded that no data were available. Eight countries (Albania, Belarus, Bulgaria, Lithuania, Moldova, Montenegro, Slovakia and Ukraine) offered no data for the review (Figure).

The contacted experts from each country gathered data from sources including national official health service reports, national databases, and international and national publications. These experts also conducted a PubMed (Medline) literature search between April and October 2016 to identify internationally published data while Google databases, using defined qualifiers for *Giardia*, *Cryptosporidium* and geographic location (e.g. Hungary), were used to identify data from grey literature. In addition, searches in local databases identified doctoral theses, journals and other publications available in the main local languages (Bosnian, Croatian, Czech, Estonian, Hungarian, Latvian, Polish, Romanian, Serbian, Slovenian) in the participating countries. Data on epidemiology, diagnostics and research of the two parasites in humans, in animals, and in the environment were extracted.

Based on the extracted data, the 95% confidence intervals (CI) of prevalence and the two-tailed p values of two-by-two table comparisons were calculated using the mid-P exact method with the OpenEpi v.3.01 programme [13]. If detailed data were not given, we report the count, percentage and CI as presented in the original publication. Data in this paper are presented on a country-by-country basis in alphabethical order.

## Results

### Bosnia and Herzegovina

#### Humans

*Cryptosporidium* spp. and *Giardia* *intestinalis* data available from routine human investigations are shown in Table 1. Investigations are performed by the Laboratory of Parasitology, Veterinary Faculty at the University of Sarajevo. Reporting on these parasites is not mandatory in Bosnia and Herzegovina.

**Table 1:** Number of laboratory-confirmed *Cryptosporidium* spp. and *Giardia intestinalis* cases^a^ and reported incidence per 100,000 inhabitants^b^, 10 countries in the eastern part of Europe, 2016

Country	Origin of stool samples^a^	Investigation method used	Investigation period	*Cryptosporidium* spp.^a^			*Giardia intestinalis* ^a^				Notifiable disease			Mean number of cases/100,000 inhabitants/year^b^		
				n positive	n total	% (95% CI)	n positive	n total		% (95% CI)	Cry		Gia	Cry	Gia	
**European Union countries**																
Croatia	Routine obligatory health checks of healthy people working with food and beverages	MIFC	2006–2015 (10 years)	–	–	–	164^c^	245,321^c^		0.07 (0.06–0.08)^c^	Yes		Yes	0.02	1.42	
	Patients with intestinal symptoms	MIFC		–	–	–	41^c^	17,183^c^		0.24 (0.17–0.32)^c^						
	Patients with bloody diarrhoea	Ziehl-Neelsen staining, microscopy		0^c^	20^c^	0.00 (0.00–13.91)^c^	–	–		–						
Czech Republic	Patients with intestinal symptoms	Different concentration and staining methods	1994–2015 (22 years)	109	NA	NA	7,926	NA		NA	Yes		Yes	0.01	0.51	
Estonia	Patients with diarrhoea	NA	2001–2015 (15 years)	6	NA	NA	5,510	NA		NA	Yes		Yes	0.05	18.28	
Hungary	Not specified	Wet mount microscopy, MIFC, ELISA, ICT	2004–2014 (11 years)	37	126,947	0.03 (0.02–0.04)	1,530	126,947		1.20 (1.15–1.27)	Yes		Yes	0.16	0.94	
Latvia	Patients with diarrhoea	ICT	2009–2015 (7 years)	57	NA	NA	–	–		–	Yes		Yes	0.29	2.48	
			2000–2015 (16 years)	–	–	–	446	NA		NA						
Poland	Not specified	NA	2007–2016 (10 years)	24	NA	NA	–	–		–	Yes		Yes	0.006	5.43	
			2005–2016 (12 years)	–	–	–	27,456	NA		NA						
Romania	Not specified	NA	2010–2015 (6 years)	–	–	–	106,682	1,870,475		5.7 (5.60–5.81)	Yes		Yes	0.01	NA	
			2008–2012 (5 years)	16	NA	NA	–	–		–						
Slovenia	Patients with diarrhoea	IFT	2002–2015 (14 years)	78^c^	5,106^c^	1.53 (1.22–1.89)^c^	–	­–		–	Yes		Yes	0.39	1.27	
	Patients with intestinal symptoms	Iodine wet mount microscopy, IFT		–	–	–	237^c^	24,782^c^		0.96 (0.84–1.08)^c^						
**Non-European Union countries**																
Bosnia and Herzegovina	Patients with diarrhoea	Flotation, IFT	2015–2016 (1.5 years)	0^c^	11^c^	0.00 (0.00–23.84)^c^	1^c^		11^c^	9.09 (0.45–37.34)^c^		No	No	NA		NA
Serbia	Routine obligatory health checks of healthy people working with food and beverages	MIFC, EIA	2004–2008 (5 years)	–	–	–	383^c^		136,334^c^	0.28 (0.25–0.30)^c^		No	Yes	NA		NA
	Patients with diarrhoea		2005–2014 (10 years)	–	–	–	1,996		NA	NA						

–: not applicable; CI: confidence interval; Cry: cryptosporidiosis; ECDC: European Centre for Disease Prevention and Control; EIA: enzyme immunoassay; Gia: giardiasis; ICT: immunochromatographic test; IFT: immunoflourescence test; MIFC: merthiolate-iodine-formaldehyde concentration; NA: not available.

^a^ Laboratory-confirmed cases as reported by national public health laboratories.

^b^ Mean notification rate extracted from 2007–2016 data in the ECDC Surveillance Atlas of Infectious Diseases [11].

^c^ Regional data, does not represent the whole country. Croatia: data from Istria region; Slovenia: data from various regions; Bosnia and Herzegovina: data from Canton of Sarajevo; Serbia: data from region of Nis.

#### Animals

At present, research on parasites is rare in Bosnia and Herzegovina and investigations have mainly focused on the presence of helminths. Some types of protozoa, such as the species of the genus *Cryptosporidium* and *Giardia,* were described as additional findings. Hodžić et al. provided the first written information about the occurrence and distribution of *Cryptosporidium* spp. and *Giardia* spp. in Bosnia and Herzegovina [14]. They investigated 123 faecal samples from red foxes (*Vulpes vulpes*) during the hunting seasons between January 2011 and March 2012. The samples were analysed for the presence of *Cryptosporidium* spp. oocysts and *G. intestinalis* cysts using sucrose flotation concentration and immunofluorescence test (IFT). *Cryptosporidium* spp. and *G. intestinalis* were detected in 3.25% (4/123; 95% CI: 1.04–7.66) and 7.32% (9/123; 95% CI: 3.63–13.00) of the samples, respectively. Co-infection with both parasites was not found. Dog faeces investigations using sucrose flotation concentration and IFT showed 5.00–6.90% positivity for *Cryptosporidium* spp. and 6.60–11.84% for *Giardia* spp. with no age-dependent differences [15,16]. However, a more recent study reported a higher prevalence (100%) of *Giardia* spp. in dogs ≤6 months of age compared with older dogs (p < 0.001) [17].

### Croatia

#### Humans

There has been an obligation for clinicians to report both parasites since 2012. However, the only data available for this review for the years 2006 to 2015 were those obtained from the Department of Microbiology, Public Health Institute of the Istrian Region, which serves an area of ca 200,000 inhabitants. Of the stool samples examined for *Giardia* cysts, 245,321 came from the obligatory occupational health checks of healthy people working with food and beverages while 17,183 were sent by clinicians for diagnostic purposes. Routine methods are used, including merthiolate-iodine-formaldehyde concentration (MIFC), to concentrate protozoa and worm eggs from faecal samples. For patients with bloody diarrhoea, Ziehl-Neelsen staining and microscopy of bloody stool are used to determine the presence of *Cryptosporidium* spp. The presence of *G. intestinalis* and *Cryptosporidium* spp. in human stool samples in the region of Istria is presented in Table 1.

### Animals

During the 9-year period from 2007 to 2015, a total of 5,387 stool samples from sick, but not necessarily diarrhoeic, canines and felines were examined for parasites at the Department for Parasitology and Parasitic Diseases with Clinic, Faculty of Veterinary Medicine, University of Zagreb. The canine and feline faecal samples were investigated by MERIFLUOR IFT (Meridian Bioscience, Inc. Cincinnati, United States (US)) after concentration by centrifugation-flotation with sucrose. *Cryptosporidium* spp. was present in 0.31% (17/5,387; 95% CI: 0.19–0.49) and *G. intestinalis* in 25.88% (1,394/5,387; 95% CI: 24.72–27.06) of canine and feline faecal samples.

### Czech Republic

#### Humans

In the years 1975 to 1982, a total of 1,750 immunocompetent persons, mostly employed by agricultural enterprises, were examined for the presence of gastrointestinal parasites [18]. Of these, none were positive for *Cryptosporidium* spp., but 0.80% (14/1,750; 95% CI: 0.48–1.38) were positive for *G. intestinalis* using Breza’s, MIFC and Army Medical Service III concentration techniques and direct microscopy [19,20]. The first human cryptosporidiosis case in the Czech Republic was recorded by Ditrich et al. in an immunodeficient patient in 1991 [21]. The authors identified the *Cryptosporidium* isolate from that case as *C. baileyi*, but the identification was made without molecular analysis and therefore cannot be considered accurate. Based on the data from National Reference Laboratory for the Diagnostics of Intestinal Parasites, Department of Parasitology, Mycology and Mycobacteriology, Prague Institute of Public Health in Ústí nad Labem, 109 findings of *Cryptosporidium* spp. and 7,926 findings of *G. intestinalis* have been reported from 1994 to 2015 across the Czech Republic (Table 1). Of these, 104 (95.41%; 95% CI: 90.13–98.30) findings of *Cryptosporidium* spp. and 5,607 (70.74%; 95% CI: 69.73–71.74) findings of *Giardia* spp. were autochthonous, while the remaining *Cryptosporidium* and *Giardia* cases represent imported infections. Few *Cryptosporidium* genotyping/subtyping results are available. In a study mapping the occurrence of various diarrhoeal pathogens in children hospitalised with diarrhoea between 1992 and 1996, 11.32% (12/106; 95% CI: 6.28–18.45) were positive for *Cryptosporidium* based on aniline-methyl-violet staining of stool smears [22,23]. Nine of 106 *Cryptosporidium*-positive samples originated from immunocompetent children 5 months to 8 years of age and were subsequently genotyped by Hajdušek et al.; eight cases of *C. parvum* and one case of *C. hominis* were reported based on PCR amplification of partial sequences of the small subunit ribosomal ribonucleic acid (SSU rRNA) and *Cryptosporidium* oocyst wall protein (COWP) genes [24]. In diarrhoeal stool samples (n = 457) from 203 immunocompetent patients under 69 years of age with suspected cryptosporidiosis, five children were positive for *C. parvum*, one child was positive for *C. hominis* and one adult was positive for *C. scrofarum* based on PCR amplification of the SSU rRNA gene [25]. Additionally, two unusual cases of cryptosporidiosis caused by *C. erinacei* and a mixed infection of *C. parvum* and *C. tyzzeri* were reported by Rašková et al. and Kváč et al. [26,27].

Seroprevalence data showed that 66.83% (133/199; 95% CI: 60.07–73.11) and 71.86% (143/199; 95% CI: 65.31–77.78) of the inhabitants of the Czech Republic have antibodies and a positive response to the 15/17-kDa and 27-kDa *Cryptosporidium* antigen groups, respectively [28]. Pospíšilová et al. showed high titres of anti-*Cryptosporidium* antibodies in 10.71% of AIDS patients (15/140; 95% CI: 6.36–16.68) [29].

#### Animals

More than 60 studies have reported the presence of *Cryptosporidium* in animals, with many including data on genotyping, host and age range, pathogenicity and host–pathogen relations. The studies also resulted in the description of *C. avium*, *C. proliferans*, *C. scrofarum*, *C. fragile*, *C. erinacei* and *C. testudinis* as novel species of the genus *Cryptosporidium* [30-46]. In the Czech Republic, *Cryptosporidium* spp. was first detected in 1979 in two 14-day old emergency slaughter bulls [47]. Until the beginning of the 21st century, *Cryptosporidium* spp. has been found in many animal hosts (cattle, goats, sheep, pigs, poultry, wild ungulates and rodents) using microscopic techniques (flotation in Sheather's sugar or Breza's solution, native preparation, aniline-carbol-methyl violet or Giemsa staining methods); however, most studies lacked genetic characterisation of the isolates [31,48-54]. In the past decade, molecular tools have been widely used to determine the species/genotype of *Cryptosporidium* present in cryptosporidiosis cases in domestic, wild and companion hosts (Table 2).

**Table 2:** Prevalence of *Cryptosporidium* species and genotypes in domestic animals including pets and wild animals using RFLP or/and sequencing of PCR products, Czech Republic, 2003–2016

Animals	n positive	n total	% (95% CI)	*Cryptosporidium* spp.	*Cryptosporidium* subtypes	Method (and sequenced molecular markers)	Reference
**Domestic**							
Cattle	11^a^	NA	NA	*C. parvum*, *C. andersoni*	NA	PCR (SSU rRNA, HSP70)	[122]
	44	995	4.42 (3.27–5.84)	*C. andersoni*, *C. parvum*, *C. bovis*	IIaA15G2R1	PCR (SSU rRNA, gp60)	[123]
	56	309	18.12 (4.12–22.72)	*C. andersoni*, *C. parvum*, *C. bovis*	IIaA16G1R1, IIaA22G1R1, IIaA18G1R1, IIaA15G1R1	PCR (SSU rRNA, gp60)	[119]
Pigs	1^a^	NA	NA	*C. suis*	NA	PCR (SSU rRNA, HSP70)	[122]
	34	123	27.64 (20.29–36.04)	*C. suis*, *C. scrofarum*, *C. parvum*	IIaA16G1R1	PCR (SSU rRNA, gp60)	[124]
	69	413	16.71 (13.34–20.54)	*C. suis*, *C. scrofarum*, *C. muris*	NA	RFLP (SSU rRNA)	[125]
	177	477	37.11 (32.86–41.51)	*C. suis*, *C. scrofarum*	NA	RFLP (SSU rRNA)	[126]
	353	1620	20.79 (7.95–16.67)	*C. suis*, *C. scrofarum*, *C. parvum*, *C. muris*	NA	RFLP (SSU rRNA)	[127]
Horses	12	352	3.41 ( 1.86–5.72)	*C. muris*, horse genotype, *C. parvum*, *C. tyzzeri*	IVaA15G4, IIaA15G2R1, IXbA22R9	PCR (SSU rRNA, gp60)	[128]
Cat	1^a^	NA	NA	*C. felis*	NA	PCR (SSU rRNA, HSP70)	[122]
Red-crowned parakeets	4^a^	NA	NA	*C. avium*	NA	PCR (SSU rRNA, HSP70, actin)	[129]
Tortoises	46	387	12.66 (8.66–15.11)	*C. testudinis, C. ducismarci,* tortoise genotype III	NA	PCR (SSU rRNA, COWP, actin)	[42]
**Wild**							
Wild boars	32	193	16.58 (11.83–22.33)	*C. suis*, *C. scrofarum*	NA	RFLP (SSU rRNA)	[130]
	61	460	13.26 (10.39–16.60)	*C. suis*, *C. scrofarum*	NA	RFLP (SSU rRNA)	[131]
Giraffe	1^a^	NA	NA	*C. muris*	NA	PCR (SSU rRNA)	[132]
Ungulates	6^a^	NA	NA	*C. ubiquitum*, *C parvum, C. andersoni*	NA	PCR (SSU rRNA, HSP70)	[122]
	10	269	3.72 (1.90–6.52)	*C. ubiquitum, C. muris*, deer genotype	XIId	PCR (SSU rRNA, gp60)	[133]
Birds	17^a^	NA	NA	*C. meleagridis, C. baileyi*	NA	PCR (SSU rRNA, HSP70)	[122]
	663^b^	NA	NA	*C. baileyi*, *C. meleagridis*	NA	PCR (HSP70)	[134]
	85^b^	NA	NA	*C. baileyi*, *C. meleagridis*	IIIeA16G2R1c	PCR (SSU rRNA, HSP70, gp60)	[135]
Mouse	14^a^	NA	NA	*C. tyzzeri*	IXaA6, IXaA8, IXbA6,	PCR (SSU rRNA, gp60, actin, COWP, TRAP-C1)	[136]
Rodents	7 ^a^	NA	NA	*C. muris*, *C. andersoni*	NA	PCR (SSU rRNA, HSP70)	[122]
Siberian chipmunks	1	1	100 (5–100)	*C. muris*	NA	PCR (SSU rRNA)	[137]
Reptiles	10^a^	NA	NA	*C. serpentis, C. varanii, C. muris*	NA	PCR (SSU rRNA, HSP70)	[122]
Rabbits	2	2	100 (22.36–100)	*C. cuniculus*	NA	PCR (SSU rRNA, HSP70)	[122]
Hedgehogs	12	15	80 (54.65–94.65)	*C. parvum, C. erinacei*	NA	PCR (SSU rRNA, gp60)	[138]
Bats	3	263	1.14 (0.29–3.07)	*C. parvum*, bat genotype III	NA	PCR (SSU rRNA)	[139]

CI: confidence interval; COWP: *Cryptosporidium* oocyst wall protein; gp60: 60 kilodalton glycoprotein; HSP70: 70 kilodalton heat shock protein; NA: not available; RFLP: restriction fragment length polymorphism; SSU rRNA: small subunit ribosomal ribonucleic acid; TRAP-C1: thrombospondin related adhesive proteins.

^a^ Selected samples.

^b^ Pooled samples.

A few studies have reported on the presence of *Giardia* in dogs, domestic animals and wild ungulates (Table 3). Unfortunately, all studies were based on microscopic examination of samples using native preparation, flotation in Sheather's sugar or Breza's solution, or staining methods. As no genotyping tools were used and information on genetic assemblages is lacking.

**Table 3:** Prevalence of *Giardia* spp. in domestic animals and wild ungulates, Czech Republic, 1993–2007

Animals	Category	n positive	n total	% (95% CI)	Reference
Dogs	Shelter	26	243	10.70 (7.26–15.07)	[140]
	Private, purebred	2	83	2.41 (0.41–7.73)	
	Private, purebred, city	37	3870	0.96 (0.68–1.30)	[141]
	Private purebred, rural area	12	540	2.22 (1.21–3.75)	
Sheep	Lamb 0.5–4 months	28	167	16.77 (11.67–23.01)	[142]
Goats	Kids 0.5–4 months	19	26	73.08 (53.86–87.39)	
Horses	Not specified	18	360	5.00 (3.08–7.64)	[143]
Roe deer	Calf 7–8 months	1	3	33.33 (1.67–86.80)	[144]

CI: confidence interval.

#### Environment

Monitoring of *Cryptosporidium* oocysts and *Giardia* cysts in drinking water resources was published by Dolejš et al. in 1999 and 2000 [55-58]. Drinking water sources in the Czech Republic have been found to contain between 0 and 32,140 *Cryptosporidium* spp. oocysts/100 L and between 0 and 485 *Giardia* spp. cysts/100 L based on IFT and microscopy. Hajdušek et al. used molecular tools to identify *C. parvum* in an open water reservoir in 2004. This isolate was recovered from 10,000 L of water using a Super Micro-Wynd 1 µm filter (CUNO Inc., Meriden, US) [24].

### Estonia

#### Humans

Cryptosporidiosis and giardiasis are notifiable diseases in Estonia. From 1991 until 2016, a total of 134 cases of cryptosporidiosis have been reported by the Health Board, of which only a few have been in the recent years (Table 1) [59,60]. For 1991 to 1992, the official Estonian reports mention 33 cryptosporidiosis cases (personal communication, J Epštein, February 2014). During the same years, stool samples of patients with intestinal diseases (n = 1,518) were examined at one hospital using an unspecified microscopy method and *Cryptosporidium* oocysts were found in 3.34% (49/1,469; 95% CI: 2.51–4.35) of the stools from patients with acute intestinal disease who were 0–14 years of age [61]. Since 1999, reports on cryptosporidiosis have originated from two of 15 counties, Harjumaa and Raplamaa, and since 2010, all the individuals diagnosed with cryptosporidiosis were children [59]. The official data thus do not appear to include known outbreaks occurring among veterinary students [62]. One such case was caused by the *C. parvum* subtype IIaA16G1R1, and there was evidence of the infection having originated from calf faeces [62].

According to the number of cases reported to ECDC from 2007­ to 2016, Estonia has the second highest rate of laboratory-confirmed giardiasis cases with a mean of 18.3 cases per 100,000 inhabitants per year (Table 1), which is three times higher than the EU mean for the same time period [11]. In particular, the reported incidence rate among children 0–4 years of age in Estonia between 2007 to 2016 (152.22 individuals per 100,000 inhabitants) was 10 times higher than the rate in all reporting countries (15.45 individuals per 100,000 inhabitants) [11]. In a national health report of the Health Board, 46.84% (549/1,172; 95% CI: 44.00–49.71) of individuals with reported giardiasis in 2010 to 2014 were children less than 5 years of age [59]. The same report reported that 5.12–17.51% of all patients with giardiasis were hospitalised and that 70.20–80.71% of the annually reported cases in 2010 to 2014 originated from one county, Harjumaa, where the capital Tallinn is located [59].

#### Animals

In 2013 to 2015, 30.04% (73/243; 95% CI: 24.53–36.03) of bovine faecal samples submitted to the veterinary and food laboratory were positive for *Cryptosporidium* spp. oocysts [63] and (personal communication, A Kärssin, February 2016). In 2010 to 2015, 5.65% (7/124; 95% CI: 2.50–10.85) of canine faecal samples submitted to diagnostic examinations but none of the 50 feline samples tested positive for *Cryptosporidium* spp [63] and (personal communication, A Kärssin, February 2016). In a cross-sectional investigation, 30.28%, (281/928; 95% CI: 27.39–33.30%) of cattle tested positive for *Cryptosporidium* spp. oocysts using a modified Ziehl-Neelsen staining [64]. The same study found 84.44% (38/45; 95% CI: 71.64–92.93) of farms to have at least one animal shedding *Cryptosporidium* spp. oocysts at the time of the study. *C. parvum* and *C. andersoni* have been described in cattle less than 12 months of age [64]. The prevalence of shedding *Cryptosporidium* spp. oocysts was higher (p < 0.001) in animals older than 12 months of age compared with younger animals. However, evaluated with a semiquantitative scale, the younger animals appeared to shed in higher numbers [64]. Management practices that appeared to increase the magnitude of oocysts shedding included early removal of a calf from its mother [65]. *Cryptosporidium* spp. oocysts were detected with IFT in ovine faeces collected from 60.87% (56/92; 95% CI: 50.63–70.43) of sheep herds on the islands of Hiiumaa, Vormsi and Saaremaa [66].

In 2010 to 2015, *Giardia* cysts were detected in 27.08% (65/240; 95% CI: 21.75–32.97) of bovine faecal samples submitted for diagnostic investigations [63], (personal communication, A Kärssin, February 2016). In the same period, 5.65% (7/124; 95% CI: 2.50–10.85) of canine faecal samples and 14.00% (7/50; 95% CI: 6.33–25.74) of feline faecal samples were positive for *Giardia* cysts [64], (personal communication, A Kärssin, February 2016). *Giardia* shedding was detected with IFT in ovine faeces collected from in 69.57% (64/92; 95% CI: 59.61–78.31) of sheep herds on the islands of Hiiumaa, Vormsi and Saaremaa [66].

### Hungary

#### Humans

Stool samples for both *Cryptosporidium* spp. oocysts and *Giardia* spp. cysts are routinely tested at the Department of Parasitology, National Center for Epidemiology and Regional Parasitological Laboratories in Budapest, Hungary using microscopic examination of the wet mount (saline and iodine) preparation, MIFC technique for concentration of the protozoan cysts, ELISA/immunochromatographic test (ICT) antigen detection and/or Kinyoun staining. The data are shown in Table 1.

Based on an epidemiological survey, the seroprevalence for a positive response to the 27-kDa *Cryptosporidium* antigen was significantly higher in communities where the drinking water originated from surface water than in the control city where riverbank filtration was used (p < 0.001). A logistic regression analysis of risk factors showed that bathing in outdoor pools was also associated with a positive response to the 15/17-kDa *Cryptosporidium* antigen complex (p = 0.0197) [67].

The association between the consumption of *Giardia*-positive drinking water and asymptomatic giardiasis was investigated in 2007. Despite this being a field investigation where only a single stool sample was examined from each participant, *G. intestinalis* infections were found in 4.00% (4/100; 95% CI: 1.28–9.36) of asymptomatic individuals. In both water samples and asymptomatic persons, *G. intestinalis* assemblage B was detected [68].

#### Animals

A total of 49.37% (39/79; 95% CI: 38.46–60.32) of faecal samples from calves with diarrhoea collected on 52 farms in 2006 from different Hungarian counties showed positivity using IFT. Based on sequence and phylogenetic analysis, *C. ryanae* was detected in one sample and the gp60 gene PCR products of 21 isolates showed that two isolates belonged to the *C. parvum* IId subtype group (IIdA22G1 and IIdA19G1) and the most common *C. parvum* subtype was IIaA16G1R1 (n = 15). Other detected subtypes were IIaA17G1R1 (n = 3) and IIaA18G1R1 (n = 1) [69].

In 2008, the combined results of a microscopic and molecular study indicated that aquatic ducks, geese, coot and cormorant may have a role in the environmental dissemination of human pathogenic assemblages of *Cryptosporidium* oocysts and *Giardia* cysts. A total of 5.82% (6/103, 95% CI: 2.39–11.72) of wild birds and 13.79% (4/29; 95% CI: 4.54–30.00) of domestic birds were *C. parvum* or *C. baileyi* positive. Additionally, 5.82% (6/103; 95% CI: 2.39–11.72) of samples from wild birds and 24.14% (7/29; 95% CI: 11.22–42.01) of samples from domestic birds were *G. intestinalis* positive [70].

In the past decade, the Central Veterinary Institute detected *Cryptosporidium* spp. in cattle, sheep and goats (Table 4).

**Table 4:** Prevalence of *Cryptosporidium* spp. in ruminants using Sheather’s sucrose flotation and direct microscopy, Hungary, 2005–2015^a^

Animal	Age category	n positive	n total	% (95% CI)
Cattle	Adult	63	7,205	0.87 (0.68–1.11)
	Post-weaned	18	466	3.86 (2.38–5.92)
	Pre-weaned	97	286	33.91 (28.60–39.55)
Sheep	Adult	1	517	0.19 (0.01–0.95)
	Post-weaned	3	175	1.71 (0.44–4.60)
	Pre-weaned	5	26	19.23 (7.41–37.60)
Goat	Adult	0	117	0.00 (0.00–2.53)
	Post-weaned	1	78	1.28 (0.06–6.94)
	Pre-weaned	2	7	28.57 (5.10–66.98)

CI: confidence interval.

^a^ Based on the database of National Food Chain Safety Office, Veterinary Diagnostic Directorate, Budapest, Hungary.

Sporadic *Cryptosporidium* spp. infections have been found by the same institute in piglets, puppies and kittens. Infection by *Giardia* spp. was detected in 27.90% of chinchillas (48/172; 95% CI: 21.59–34.96). Sporadic *Giardia* spp. infections were also seen in cattle, sheep, dogs, cats and laboratory rats. The presence of *G. intestinalis* in kennel dogs from Hungary using a specific copro-antigen ELISA test was 58.82% (110/187; 95% CI: 51.66–65.72). All sequenced SSU rRNA samples belonged to dog-specific assemblages C and D. Although canine giardiasis is highly prevalent in the studied geographical areas, it did not present zoonotic potential and the infection rate declined with increasing age of the dogs [71].

#### Environment

The presence of *Cryptosporidium* oocysts and *Giardia* cysts in different water sources (surface water, wastewater, raw water and drinking water) was investigated during the period 2000 to 2007 by microscopy using Method 1623 of the United States Environmental Protection Agency (US EPA). Up to three *Cryptosporidium* oocysts/100 L and up to 63.6 *Giardia* cysts/100 L were detected in drinking water [72]. The highest concentration in raw water was 50 *Cryptosporidium* oocysts/100 L and 1,030 *Giardia* cysts/100 L. A higher concentration of oocysts was found in water sources that received effluents from sewage treatment plants or originated from a forest environment. Riverbank filtrated water (n = 71) and raw water from the Danube River (n = 184) in Budapest were monitored to document the protozoan removal efficiency by riverbank filtration (RBF) during the years 2004 to 2005 [72] and (Plutzer et al. data not shown). *Cryptosporidium* and *Giardia* spp*.* were detected regularly in the river water but never in riverbank filtered water, suggesting the effectiveness of RBF as a method of pathogen removal. *Cryptosporidium* spp. were detected in 36.41% of raw river water samples (67/184; 95% CI: 29.70–43.55) and *Giardia* spp. were detected in 96.74% of raw river water samples (178/184; 95% CI: 93.34–98.67) [72] and (Plutzer et al. data not shown). The species and genotypes determined by molecular tools were all potentially zoonotic: *C. parvum*, *C. meleagridis* and *G. intestinalis* assemblages A and B [72,73].

### Latvia

#### Humans

The epidemiological data regarding *Cryptosporidium* and *Giardia* were collected from the Centre of Disease Prevention and Control of Latvia (Table 1). Cryptosporidiosis cases in humans have only been reported since 2009, with a total of 57 cases being reported from then until 2015 (mean: 9 cases per year, range: 2–23 cases per year). The highest number of reported cases occurred in the age group of 30–39 years olds: 42.11% (24/57; 95% CI: 29.83–55.16).

From 2000 to 2015, a total of 446 cases of giardiasis were reported (mean: 30 cases per year, range: 3–124 cases per year). The highest number of reported cases, 30.94% (138/446; 95% CI: 26.78–35.35), was observed in the age group of 7–14 year olds. All diagnostics were conducted by analysing stool samples with the copro-antigen test.

#### Animals

During a study conducted by the Faculty of Veterinary Medicine at the Latvia University of Agriculture between 2013 and 2014, a total of 1,580 faecal samples from dairy cattle were collected from different regions in Latvia. According to the microscopy results using Ziehl-Neelsen staining, *Cryptosporidium* oocysts were present in 19.43% (307/1,580; 95% CI: 17.54–21.44) of the samples. A lower prevalence of *Cryptosporidium* spp.-positive faecal samples was found 4.64% (18/388; 95% CI: 2.86–7.09) in the Latgale region than in other regions where prevalence ranged from 20.39% (63/309; 95% CI: 16.17–25.16) to 26.38% (86/326; 95% CI: 21.81–31.37). An earlier study of 16 dairy farms and 125 animals found that 68.75% (11/16; 95% CI: 43.68–87.54) of the farms had at least one animal shedding *Cryptosporidium* spp. in their faeces; 40.80% (51/125; 95% CI: 32.44–49.58) of the animals tested positive using modified Ziehl–Neelsen staining of faecal smears [74]. There are no *Cryptosporidium* prevalence studies in other animal species. No studies have investigated the prevalence of giardiasis in animals, while sporadic *G. intestinalis* infections are diagnosed in dogs and cats (personal communication, G Deksne, July 2016).

### Poland

#### Humans

In Poland, human cryptosporidiosis and giardiasis cases are notifiable diseases. In the years 2005 to 2016, the National Institute of Public Health, National Institute of Hygiene in Poland reported one to six cases of human cryptosporidiosis and 2,288 cases of giardiasis per year (Table 1). Prevalence estimates of *Cryptosporidium* and *Giardia* spp. in humans are available from research studies, but these are limited to selected population groups and regions. For example, *Cryptosporidium* oocysts were detected by microscopy (examination of smears of faecal samples after modified Ziehl-Neelsen and IFT) in 14.63% (36/246; 95% CI: 10.62–19.47) of stool samples collected from hospitalised patients with diarrhoea [75]. All positive samples were from children up to 4 years of age, and isolates belonged to species *C. parvum* and *C. hominis*. In 2008, Bajer et al. reported *Cryptosporidium* infections in persons with immunodeficiencies; *C. hominis*, *C. meleagridis* and *C. parvum* were found in children with primary immunodeficiencies (PID), but only *C. parvum* was found in children and adults with a secondary immunosuppression (i.e. after cancer treatment) [76].

A 2010 study including 232 people from the west-central region of Poland found *G. intestinalis* in 1.29% (3/232; 95% CI: 0.33–3.48) of the collected faecal samples by direct microscopy. Three subgenotypes of *Giardia* were detected: a cosmopolitan subgenotype AII and two new subgenotypes A and B [77]. Examination of the faeces of 31,504 children 7 years of age from 15 Polish provinces in 2002 to 2003 found *G. intestinalis* in the faeces of 0.69% (217/31,504; 95% CI: 0.60–0.78) of the children using direct microscopy and Lugol’s iodine staining method [78]. In another study from 2008 to 2009, of 120 children with watery diarrhoea resembling a parasite infection, 12.50% (15/120; 95% CI: 7.44–19.35) tested positive for *Giardia* antigens in the faeces using an immunochromatographic test [79].

#### Animals

Several prevalence studies have been performed on animals in Poland for both parasites using a wide range of detection techniques. The results are summarised in Table 5.

**Table 5:** Prevalence of *Cryptosporidium* spp. and *Giardia* spp. in domestic animals including pets and wild animals using different methods Poland, 1997–2014

Animals	n positive	n total	% (95% CI)	*Cryptosporidium* spp.	*Giardia* spp.*/G. intestinalis* assemblage	Method (and sequenced molecular markers)	Reference
**Domestic**							
Cattle	10	86	11.63 (6.06–19.75)	NE	*G. intestinalis/A, E*	PCR (β-giardin)	[145,146]
	16	86	18.60 (11.42–27.87)	NE	*Giardia* spp.	IFT	
	119	700	17.00 (14.35–19.92)	*C. bovis, C. parvum, C. andersoni, C. ryanae*	NE	PCR (SSU rRNA, COWP)	[147]
Pigs	8	84	9.52 (4.52–17.28)	NE	*G. intestinalis* / B, E	PCR (β-giardin)	[145,146]
	25	84	29.76 (20.73–40.17)	NE	*Giardia* spp.	IFT	
	46	166	27.71 (21.31–34.89)	*C. scrofarum, C. suis, C. parvum*	NE	PCR (SSU rRNA, COWP)	[148]
Horses	1	10	10 (0.50–40.35)	NE	*G. intestinalis* / E	PCR (β-giardin)	[145,146]
	1	10	10 (0.50–40.35)	NE	*Giardia* spp.	IFT	
	20	564	3.55 (2.24–5.33)	*C. parvum*	NE	EIA, IFT, FISH	[149]
Sheep	18	81	22.22 (14.17–32.23	NE	*G. intestinalis* / A, E	PCR (β-giardin)	[145,146]
	17	81	20.99 (13.16–30.86)	NE	*Giardia* spp.	IFT	
	16	159	10.06 (6.07–15.50)	*C. parvum*	NE	Microscopy^a^	[150]
Goats	0	46	0.00 (0.00–6.31)	*Cryptosporidium* spp.	NE	Microscopy^a^	
Cats	4	160	2.50 (0.80–5.92)	NE	*G. intestinalis*/A, B	PCR (GDH)	[151]
Dogs	3	60	5.00 (1.29–13.00)	NE	*G. intestinalis*/A, E	PCR (β-giardin)	[145,146]
	7	60	11.67 (5.25–21.72)	NE	*Giardia* spp.	IFT	
	32	350	9.14 (6.45–12.51)	NE	*G. intestinalis*/A, C, D	PCR (GDH)	[152]
	18	350	5.14 (3.28-7.28)	NE	*G. intestinalis*	Microscopy^a^	
	2	148	1.35 (0.23–4.39)	NE	*G. intestinalis*/C, D	PCR (β-giardin)	[153]
	8	64	12.5 (5.98–22.36)	*Cryptosporidium* spp.	NE	IFT	[154]
	23	64	35.94 (24.92–48.20)	NE	*Giardia* spp.		
Domestic birds	1	101	0.99 (0.50–4.79)	NE	*G. intestinalis*	Microscopy^a^, FISH	[155]
	0	101	0.00 (0.00–2.92)	*C. parvum*	NE	Microscopy^a^, EIA, FISH	
**Wild**							
Wild boars	4	27	14.81 (4.89–31.97)	NE	*Giardia* spp.	IFT	[145,146]
	11	27	40.74 (23.62–59.76)	NE	*G. intestinalis* / B	PCR (β-giardin)	
	0	5	0.00 (0.00–45.07)	*Cryptosporidium* spp.	NE	Microscopy^a^, IFT, PCR (COWP)	[156]
	0	5	0.00 (0.00–45.07)	NE	*Giardia* spp.	Microscopy^a^, IFA	
Foxes	0	21	0.00 (0.00–13.29)	NE	*G. intestinalis*	PCR (β-giardin)	[145,146]
	4	21	19.05 (6.36–39.80)	NE	*Giardia* spp.	IFT	
Red deer	5	28	17.86 (6.85–35.24)	NE	*G. intestinalis*/B	PCR (β-giardin)	
	0	28	0.00 (0.00–10.15)	NE	*Giardia* spp.	IFT	
	14	52	26.92 (16.22–40.14)	*Cryptosporidium* spp.	NE	Microscopy^a^, IFT, PCR (COWP)	[156]
	1	52	1.92 (0.10–9.12)	NE	*Giardia* spp.	Microscopy^a^, IFT	
	1	61	1.64 (0.08–7.82)	NE	*Giardia* spp.	Microscopy^a^	[157]
Roe deer	11	48	22.92 (12.68–36.33)	NE	*G. intestinalis*/B	PCR (β-giardin)	[145,146]
	2	48	4.17 (0.70–13.09)	NE	*Giardia* spp.	IFT	
	2	22	9.09 (1.55–26.92)	*Cryptosporidium* spp.	NE	Microscopy^a^, IFT, PCR (COWP)	[156]
	1	22	4.55 (0.23–20.44)	NE	*Giardia* spp.	Microscopy^a^, IFT	
	2	50	4.00 (2/50; 0.68–12.59)	NE	*Giardia* spp.	Microscopy^a^	[157]
Fallow deer	0	65	0.00 (0/65; 0.00–4.50)	NE	*Giardia* spp.		
Moose	0	5	0.00 (0/5; 0.00–45.07)	NE	*Giardia* spp.		
	4	23	17.39 (4/23; 5.78–36.80)	NE	*G. intestinalis*	PCR (β-giardin)	[145,146]
	0	23	0.00 (0/23; 0.00–12.21)	NE	*Giardia* spp.	IFT	
Wolves	2	7	28.57 (5.10–66.98)	NE	*G. intestinalis*/D	PCR (β-giardin)	
	2	7	28.57 (5.10–66.98)	NE	*Giardia* spp.	IFT	
	5	14	35.71 (14.44–62.40)	*C. parvum*, genotype 2	NE	Microscopy^a^, IFT, PCR (COWP)	[156]
Rodents	10^b^	266	NA	NE	*G. microti, G. muris*	PCR (SSU rRNA)	[158]
	8^b^	266		*C. parvum, C. ubiquitum*	NE		
	41	114	35.9 (27.74–45.54)	*Cryptosporidium* spp.	NE	IFT	[159]
	0	114	0.00 (0.00–4.06)	NE	*Giardia* spp.		
	NA	NA	28.1–62.3 (NA)	*Cryptosporidium* spp.	NE	Microscopy^a^, IFT	[160]
	NA	NA	24.4–74.2 (NA)	NE	*Giardia* spp.		
European beaver	7	22	31.82 (15.11–53.05)	*Cryptosporidium* spp.	NE	Microscopy^a^, IFT, PCR (COWP)	[156]
	1	22	4.55 (0.23–20.44)	NE	*Giardia* spp.	Microscopy^a^, IFT	
European bison	16	55	29.09 (18.27–42.07)	*Cryptosporidium* spp.	NE	Microscopy^a^, IFT, PCR (COWP)	
	4	55	7.27 (2.35–16.62)	NE	*Giardia* spp.	Microscopy^a^, IFT	
Polish Konik (horse)	0	10	0.00 (0.00–25.89)	*Cryptosporidium* spp.	NE	Microscopy^a^, IFT, PCR (COWP)	
	1	44	2.27 (0.11–10.70)	*Cryptosporidium* spp.	NE	Microscopy^a^	[161]
Birds captive	2	90	2.22% (0.37–7.15)	NE	*G. intestinalis*	Microscopy^a^, FISH	[155]
	1	90	1.11% (0.06–5.36)	*C. parvum*	NE	Microscopy^a^, EIA, FISH	

COWP: *Cryptosporidium* oocyst wall protein; EIA: enzyme immunoassay; IFT: immunofluorescent test; FISH: fluorescent in situ hybridisation; GDH: glutamate dehydrogenase; NA: not available; NE: not examined; SSU rRNA: small subunit ribosomal ribonucleic acid.

^a^ Examination of smears of faecal samples or samples after Sheather’s flotation and after modified Ziehl-Neelsen or Lugol’ iodine or iron hematoxylin staining, or Willis-Schlaf or McMaster methods.

^b^ Number of sequenced samples.

#### Environment

*Cryptosporidium* spp. contamination of tap water has been confirmed by microscopy, IFA and PCR in one of twelve examined samples from the city of Poznan [80].

##### Examination of surface waters

The presence of *G. intestinalis* assemblages A and B, and *Cryptosporidium* oocysts has been found in 45.57% (36/79; 95% CI: 34.84–56.61) and 32.91% (26/79; 95% CI: 23.24–43.82) of samples taken from Mazurian Lake, respectively [81]. The Vistula River (n = 21) and the Zegrzyński Lake (n = 8) were tested for the presence of *Cryptosporidium* oocysts and *Giardia* cysts using a Filta-Max filtration capsules and xpress automatic station (IDEXX Laboratories, Inc., Westbrook, US) for filter elution, immunomagnetic separation (IMS) and IFT [82]. *Giardia* cysts were found in all samples from the Zegrzynski Lake (range: 10–45/100 L) and in all samples from the Vistula River (range: 10–389/100 L). *Cryptosporidium* oocysts were present in 50.00% (4/8; 95% CI: 18.41–81.59) of samples from the Zegrzyński Lake and in 47.62% (10/21; 95% CI: 27.29–68.57) of samples from the Vistula River. Their number in both cases was similar and ranged from 5 to 25 oocyst/100 L. *Cryptosporidium* oocysts were also detected in 50 of 68 surface water samples collected monthly from intakes (n = 13) and recreational waters (n = 4) in the Krakow area during June to September 2012. *Giardia* cysts were only detected in samples taken from three sampling locations [83].

##### Examination of sewage waters

*Cryptosporidium* spp. oocysts were detected in 61.54% (8/13; 95% CI: 34.09–84.32) of wastewater treatment plants (WWTPs) and *Giardia* spp. cysts in 84.61% (11/13; 95% CI: 57.77–97.34) of WWTPs in eastern Poland by microscopic analyses using Method 1623 of the US EPA [84]. *Cryptosporidium* oocyst concentrations in raw sewage water ranged from 40 to 15,410 oocysts/100 L and *Giardia* cyst concentrations ranged from 70 to 66,000 cysts/100 L.

##### Using animals as indicators of contamination

Rotifers taken from three lakes located near the city of Poznań were used as an indicator of recreational water contamination [85]. *Cryptosporidium* oocysts were detected in rotifers and water from the lakes using the fluorescence in situ hybridisation (FISH) method. Mussels collected from Poznań’s municipal reservoir, Lake Malta, have been examined by direct microscopy (wet smear and smears stained with Ziehl–Neelsen and iron haematoxylin) and MERIFLUOR IFT *Cryptosporidium*/*Giardia* kit (Meridian Bioscience Inc., Cincinnati, US) [86]. *Cryptosporidium* oocysts were detected in 15.38% (12/78; 95% CI: 8.61–24.69) of the mussels.

##### Contamination of food products

Fresh vegetables and soft fruit have been investigated using IMS and molecular methods [87]. *Cryptosporidium* oocysts were found on 6 of 128 vegetables, and *C. parvum* was identified by subtyping (gp60) from celery. The authors speculated that the presence of *Cryptosporidium* on vegetables could be associated with products originating from regions with considerable livestock production [87].

### Romania

#### Humans

Between 2008 and 2012, a total of 16 *Cryptosporidium* spp. infections were reported by the Romanian National Public Health Institute (Table 1). In a study using ELISA, a *Cryptosporidium* prevalence of 4.04% (17/421; 95% CI: 2.45–6.26) was reported from western Romania [88]. Molecular characterisation of five isolates indicated the presence of species *C. parvum* (n = 3) and *C. ubiquitum* (n = 2) [88]. Vieira et al. has also reported the presence of the *C. parvum* subtype IIdA22G1 in faecal samples of four children under 12 years of age from Timiş County in this area of Romania [89].

Data provided by the Romanian National Public Health Institute for a 6-year period (2010 to 2015) of routine investigation of patients with gastrointestinal disorders showed a cumulative *Giardia* infection prevalence of 5.70% (106,682/1,870,475; 95% CI: 5.60–5.81). Data from 269 hospitalised patients between 1996 and 2008 from Caraş-Severin County indicated *Giardia* infections in 7.81% (21/269; 95% CI: 5.03–11.49) of individuals [90]. In addition, a study conducted by Costache et al. between 2008 and 2011 in Cluj County and neighbouring areas reported a cumulative giardiasis prevalence of 0.41% (76/18,486; 95% CI: 0.33–0.51) in children and of 0.80% (141/17,645; 95% CI: 0.67–0.94) in the adult general population [91].

#### Animals

Over the last decade, epidemiological surveys were carried out with the aim of finding *Cryptosporidium* oocysts and *Giardia* cysts in livestock, pets and wildlife stool samples. Research focusing on livestock is limited and mostly involves the western [89,92-94], central and north-western [95] regions of the country. The methods applied included non-molecular (conventional acid-fast staining and classical microscopic examination, copro-antigen detection immunoassays) and molecular tools (PCR-restriction fragment length polymorphism (RFLP), DNA sequencing). Results are summarised in Table 6.

**Table 6:** Prevalence and identification of *Cryptosporidium* spp. subtypes and *Giardia intestinalis* assemblages in animals using non-molecular and molecular methods, Romania, 2005–2016

Animals	Non-molecular				Molecular^a^			References
	n positive	n total	% (95% CI)	Methods used	Species identified	*Cryptosporidium* subtypes/*Giardia* assemblages (number of specimens)	Method (and sequenced molecular markers)	
***Cryptosporidium***								
Cattle	65	258	25.19 (20.18–30.76)	Microscopy after mZN staining	*C. parvum*	IIaA15G2R1 (8), IIaA16G1R1 (7)	PCR (SSU rRNA, gp60)	[92]
	198	708	27.97 (24.75–31.36)	Microscopy after mZN staining	–	–	–	[95]
	–	–	–	–	*C. parvum*	IIdA27G1 (8), IIdA25G1 (5), IIdA22G1 (2), IIdA21G1a (1), IIaA16G1R1 (1)	PCR (SSU rRNA, gp60)	[89]
Lambs	24	175	13.71 (9.20–19.42)	Microscopy after mZN staining	*C. parvum* (83.4%), *C. ubiquitum* (8.3%), *C. xiaoi* (8.3%)	IIaA17G1R1 (2), IIaA16G1R1 (2), IIdA20G1 (2), IIdA24G1 (1), IIdA22G2R1 (1)	PCR (SSU rRNA, gp60)	[93]
Goat kids	99	412	24.03 (20.09–28.33)	Microscopy after mZN staining	–	–	–	[95]
Pigs	–	–	–	–	*C. parvum*	IIdA26G1	PCR (SSU rRNA, gp60)	[89]
***Giardia***								
Cattle	239	621	38.49 (34.72–42.36)	ELISA	–	–	–	[94]
Lambs	432	615	70.24 (66.54–73.76)	ELISA	–	–	–	
Dogs	52	614	8.47 (6.46–10.87)	Direct microscopy after flotation	–	–	–	[162]
	114	416	27.40 (23.28–31.84)	ELISA	–	–	–	
	102	215	47.44 (40.82–54.13)	ELISA	–	–	–	[94]
	–	–	–	–	*G. intestinalis*	D (29), C (8), E (1), C and D (1)	PCR (GDH)	[163]
	–	–	–	–	*G. intestinalis*	D (8), C (8)	PCR (SSU rRNA)	[111]
Cats	3	414	0.72 (0.18–1.96)	Direct microscopy after flotation	–	–	–	[164]
	51	183	27.87 (21.74–34.70)	ELISA	–	–	–	[165]
	66	264	25.00 (20.06–30.49)	Direct microscopy after Lugol’s iodine staining	–	–	–	[94]
	–	–	–	–	*G. intestinalis*	D	PCR (GDH)	[163]
Wolves	–	–	–	–	*G. intestinalis*	D	PCR (GDH)	[163]
Muskrat	–	–	–	–		C		
Raccoon dog	–	–	–	–		D		
Roe deer	–	–	–	–		E		
Fallow deer	–	–	–	–		E		

–: not applicable; CI: confidence interval; ELISA: enzyme-linked immunosorbent assay; GDH: glutamate dehydrogenase; gp60: 60 kilodalton glycoprotein; mZN: modified Ziehl-Neelsen; SSUrRNA: small subunit ribosomal ribonucleic acid.

^a^ For molecular studies microscopically positive samples have been used.

#### Environment

Investigations on the occurrence of *Cryptosporidium* spp. oocysts and *Giardia* spp. cysts in the main rivers of western Romania using the US EPA’s Method 1623 showed their presence in 7.54% (4/53; 95% CI: 2.44–17.21) and 41.50% (22/53; 95% CI: 28.87–55.06) of raw surface water samples, respectively. Genetic characterisation of the isolates demonstrated the presence of domestic/wild canid origin *C. canis* (n = 1) and the human/animal origin *C. parvum* IIaA16G1R1 subtype (n = 1), as well as *G. intestinalis* assemblages AII (n = 12) and E, the ruminant origin assemblage (n = 1) [96]. In another study, conducted in the same region, 27.27% (3/11; 95% CI: 7.45–57.81) of the tested wastewater samples were positive for the zoonotic *C. parvum*, with IIaA15G2R1 (n = 2) and IIdA18G1 subtypes. Also, the occurrence of *Giardia* spp. were recorded in different surface water types with a detection rate of 90.91% (10/11; 95% CI: 62.66–99.55) in wastewaters, 26.31% (5/19; 95% CI: 10.34–49.06) in brooks, 37.50% (3/8; 95% CI: 10.56–72.20) in irrigation channels, 31.25% (5/16; CI: 12.46–56.32) in lakes, and 36.36% (8/22; CI: 18.53–57.59) in ponds. The registered and successfully sequenced *G. intestinalis* assemblages were: assemblage E (n = 12) in all tested water bodies, assemblage AII (n = 9) in all tested water bodies except for ponds, and the domestic/wild canid specific assemblage D in a pond [97].

### Serbia

#### Humans

In Serbia, giardiasis is a notifiable disease, while cryptosporidiosis is not. Not only that cryptosporidiosis is not reportable, it has also seldom been the subject of research. The only description of cryptosporidiosis in immunocompetent individuals is a report of a family outbreak in 2010 [98]. Conversely, a long-term analysis in immunocompromised individuals carried out between 1985 and 2008 found cryptosporidiosis in 10.50% (50/476; 95% CI: 7.98–13.50) of HIV-infected patients with gastrointestinal symptoms. This finding placed cryptosporidiosis as the second most common cause of gastrointestinal disorders, following oesophageal candidiasis, among all opportunistic diseases in this patient category [99].

On the other hand, giardiasis apparently occurs much more frequently. From 2005 to 2014, a total of 1,996 cases of giardiasis (Table 1) were reported by the Institute of Public Health of Serbia [100]. However, the number of examinations carried out, the clinical reasons for testing and the methods used in particular laboratories are not reported. Analysis of the reports showed that the number of reported cases of giardiasis decreased from 4.6 per 100,000 inhabitants in 2005 to 1.1 per 100,000 inhabitants in 2014. There was no difference (p = 0.255) in the distribution of cases between females and males (48.5% of cases were female and 51.5% were male). Infections were most often diagnosed in people aged 20–40 (45.6%), while 11.9% of all cases were reported in children up to 10 years of age. Giardiasis occurrence was associated with seasonality (p < 0.0001), with one third of the cases being diagnosed between August and October. The incidence peak coincided with increased outdoor activities and increased water consumption during hot weather periods. Giardiasis is widespread throughout Serbia, but the data seem to indicate that it is more common in northern than in central Serbia (10-year mean of 4.5 cases/100,000 inhabitants vs 2.1 cases/100,000 inhabitants). Whether the observed fluctuations reflect a real change in the infection dynamics or are merely the result of differences in the detection of cases or reporting of these remains to be explored. Official reports do not differentiate between cases and do not describe whether reported cases were symptomatic or accidental findings of possibly asymptomatic individuals, for example, during routine examinations of cooks, bakers, restaurant staff, etc. for obligatory occupational health checks. Regional investigations conducted by the Department for Parasitology at the Public Health Centre of Niš (southern Serbia) between 2004 and 2008, did report the number of investigations making it possible to estimate the prevalence of *Giardia*, which was 0.28% (Table 1). Miladinovic-Tasic and colleagues carried out several studies on giardiasis in different populations; the results from ones that examined healthy adults as a part of obligatory occupational health checks showed a decrease in the prevalence of giardiasis from 0.43% (64/14,833; 95% CI: 0.33–0.55) in 2002 to 0.16% (53/32,814; 95% CI: 0.12–0.21) in 2008 [101-103]. High infection rates were registered in establishments where people were in close contact, such as individuals in psychiatric institutions (6/100; 6.00%, 95% CI: 2.47–12.06) [101], specialised institutions for children with disabilities (7/106; 6.60%, 95% CI: 2.93–12.62) [101] and refugee camps (7/122; 5.74%, 95% CI: 2.54–11.02) [102]. In patients with diarrhoea, the prevalence of giardiasis was as high as 10% in adults and 4% in children under 14 years of age [101,102]. The prevalence of giardiasis has also been studied in schoolchildren. Nikolić et al conducted an extensive long-term study throughout central Serbia between 1985 and 2005 that involved a total of 6,645 asymptomatic children 7–11 years of age, representing approximately 10% of the total age-matched population (n = 69,232) [104]. The methods used included microscopy after conventional concentration techniques. Despite this being a field investigation where only a single stool sample was examined from each participant, the results showed the presence of *Giardia* infection in all examined regions, with infection rates ranging from 3.2 to 14.2%, and an overall prevalence of 6.10% (405/6,645; 95% CI: 5.54–6.69). This is significantly higher than the figures in the official reports. Interestingly, the prevalence of *Giardia* was similar in urban (7.0%) and rural (6.5%) areas. Another study had previously shown a similarly high prevalence of 8.00% (14/175; 95% CI: 4.63–12.76) in the highly urban area of the city of Belgrade [105]. Finally, a study carried out in 2004 in south-western Serbia estimated a giardiasis prevalence of 5.62% (45/800; 95% CI: 4.18–7.39) in asymptomatic schoolchildren [106].

#### Animals

Neither *Cryptosporidium* nor *Giardia* infections are notifiable in animals in Serbia. However, several studies have investigated such infections in cattle, swine, lambs and goats (Table 7).

**Table 7:** Prevalence of *Cryptosporidium* spp. and *Giardia intestinalis* and genotypes in animals using microscopy^a^ and PCR, Serbia, 2002–2015

Animals	n positive	n total	% (95% CI)	*Cryptosporidium* subtypes/*Giardia* assemblages (number of specimens)	Method (and sequenced molecular markers)	References
***Cryptosporidium***						
Cattle	72	160	45.00 (37.30–52.90)	NA	Microscopy	[107]
	62	103	60.19 (50.52–69.30)	*C. parvum* IIa (10), IIaA16G1R1b, IIaA18G1R1 IIa A20G1R1, IId (2), IId A18G1b, IIj (6), IIjA16R2, IIjA17R2	PCR (SSU rRNA, COWP)	[108]
	6	30	20.00 (8.53–37.03)	NA	Microscopy	[166]
Swine	89	260	34.23 (28.65–40.16)	NA	Microscopy	[109]
	14	34	41.18 (25.69–58.11)	NA	Microscopy	[166]
Lambs	53	126	42.06 (33.67–50.82)	NA	Microscopy	[167]
	12	25	48.00 (29.19–67.25)	NA	Microscopy	[166]
Goat	28	88	31.82 (22.74–42.08)	NA	Microscopy	[167]
***Giardia***						
Dogs	22	151	14.57 (9.60–20.89)	NA	Microscopy	[110]
	88	134	65.67 (57.33–73.34)	*G. intestinalis* C (8), D (6)	PCR (SSU rRNA)	[111]
Cats	18	81	22.22 (14.17–32.23)	NA	Microscopy	[112]
	6	50	12.00 (5.01–23.29)	NA	Microscopy	[113]

CI: confidence interval; COWP: Cryptosporidium oocyst wall protein; EIA: enzyme immunoassay; SSU rRNA: small subunit ribosomal ribonucleic acid; NA: not available.

^a^ Microscopy: examination of faecal sample smears or fecal samples after Sheather’s flotation and modified Ziehl-Neelsen, Lugol’ iodine or iron hematoxylin staining.

In an examination of 160 cattle from the Belgrade area, *Cryptosporidium* oocysts were detected in 34.61% (9/26; 95% CI: 18.38–54.11) of weaners, 49.02% (25/51; 95% CI: 35.55–62.60) of bull calves and 47.50% (38/80; 95% CI: 36.74–58.44) of post parturient cows [107]. Another study showed a prevalence of 60.20% (62/103; 95% CI: 50.52–69.30) among dairy calves up to 1 month of age [108]. *Cryptosporidium* oocysts were also found in the faeces of 34.23% (89/260; 95% CI: 28.65–40.16) of swine with an observed decrease with age [109]. To expand, oocysts were detected in 45.55% (41/90; 95% CI: 35.49–55.91) of nursing, weaning and post-weaned piglets up to 3 months of age, in 32.80% (41/125; 95% CI: 25.00–41.39) of post-weaned piglets 3 to 12 months of age, and in 15.55% (7/45; 95% CI: 7.07–28.36) of sows older than 12 months of age. In all pigs older than 3 months of age, the *Cryptosporidium* infection was subclinical [109].

Infections with *Giardia* were studied in 2008 in Belgrade-area dogs and cats (Table 7). In dogs, the infection rate depended on living conditions. The lowest prevalence was detected in pet household dogs (7.41%, 6/81; 95% CI: 3.06–14.77), followed by a higher prevalence in stray (18.67%, 14/75; 95% CI: 11.04–28.68) and kennel dogs (36.36%, 4/11; 95% CI: 12.78–66.36) [110]. A 2015 study in shelter dogs, however, showed a remarkably higher prevalence of infections with *Giardia* of 65.67% (88/134; 95% CI: 57.33–73.34) belonging to the assemblages C and D [111]. In 2001, a study found a higher *Giardia* prevalence in kittens (7/23; 30.43%, 95% CI: 14.39–51.14) than in adult pet cats (11/58; 18.96%, 95% CI: 10.40–30.57), however all 95% CIs overlapped [112]. Other data from 2012 seemed to indicate a decrease in the *Giardia* infection rate in cats [113].

### Slovenia

#### Humans

Between 2002 and 2015, patients with diarrhoea (n = 5,106) were examined for *Cryptosporidium* oocysts by IFT and patients with various gastrointestinal and/or digestive disorders and/or diseases (n = 24,782) were examined for the presence of *G. intestinalis* cysts by iodine wet mount microscopy and/or IFT at the Institute of Microbiology and Immunology (IMI), Faculty of Medicine at the University of Ljubljana. It was found that 78/5,106 (1.53%) and 237/24,782 (0.96%) patients were *Cryptosporidium* and *G. intestinalis* positive, respectively (personal communication, B Šoba, November 2016) (Table 1). In the same period (2002–2015), 121 cases of cryptosporidiosis and 574 cases of giardiasis were reported to the National Institute of Public Health of the Republic of Slovenia (NIJZ), with a mean cryptosporidiosis and giardiasis incidence of 0.42 and 2.02 per 100,000 inhabitants in Slovenia, respectively [114]. *Cryptosporidium* species and subtypes identified from human samples are summarised in Table 8.

**Table 8:** *Cryptosporidium* spp. and subtypes detected in faecal samples from humans and cattle using PCR, Slovenia, 2000–2015

Type of sample	Time period	*Cryptosporidium* spp. (number of specimens)	*Cryptosporidium* subtypes (number of specimens)	Method (and sequenced molecular markers)	Reference
Human	2000–2006	*C. hominis* (2)	IaA17 (1), IbA10G2 (1)	PCR (SSU rRNA, gp60)	[117,168]
		*C. parvum* (31)	IIaA9G1R1 (1), IIaA11G2R1 (2), IIaA13R1 (2), IIaA14G1R1 (1), IIaA15G1R1 (4), IIaA15G2R1 (15), IIaA16G1R1 (2), IIaA17G1R1 (1), IIaA19G1R1 (1), IIcA5G3 (1), IIlA16R2 (1)		
		*C. ubiquitum* (1)	NA		
Human	2007–2015	*C. hominis* (7)	IaA20 (1), IaA22 (1), IaA23 (1), IdA14 (1)	PCR (SSU rRNA, gp60)	(Šoba et al. data not shown)
		*C. parvum* (32)	IIaA11R1 (1), IIaA13R1 (10), IIaA15G2R1 (14), IIaA15G1R1 (2), IIaA16R2 (1), IIaA16G1R1 (1), IIaA17G1R1 (1), IIaA19G1R1 (1)		
		*C. meleagridis* (1)	NA		
Bovine	2002–2007	*C. parvum* (45)	IIaA13R1 (5), IIaA15G2R1 (27), IIaA16R1 (3), IIaA16G1R1 (6), IIlA16R2 (2), IIlA18R2 (2)	PCR (SSU rRNA, gp60)	[117]
		*C. bovis* (3)	NA		
		*C. ryanae* (3)	NA		

NA: not available: SSU rRNA: small subunit ribosomal ribonucleic acid.

From 2002 to 2013, a total of 51 *G. intestinalis* isolates from symptomatic human cases were genetically characterised. Assemblage A was found in 50.98% (26/51; 95% CI: 37.40–64.45) of the isolates while the remaining 49.02% (25/51; 95% CI: 35.55–62.60) of the isolates were of the assemblage B. Phylogenetic analysis showed that the successfully subtyped assemblage A isolates belonged to the sub-assemblage AII while the assemblage B isolates belonged to the sub-assemblage BIV [115].

#### Animals

According to genotyping studies, the transmission of *Cryptosporidium* between cattle and humans is of epidemiological relevance in Slovenia. The most common *C. parvum* subtypes in cattle were also found in humans [116,117]. The *Cryptosporidium* species and subtypes detected in cattle in Slovenia are presented in Table 8.

Faecal samples from cattle (n = 391), sheep (n = 35), goats (n = 9), horses (n = 14) and deer (n = 28), were examined for *Giardia* cysts using IFT in 2006–2007. Of the examined samples, 26.60% (104/391; 95% CI: 22.40–31.15) of cattle, 42.86% (15/35; 95% CI: 27.35–59.50) of sheep and 11.11% (1/9; 95% CI: 0.55–43.86) of goats were found to be *Giardia*-positive, while no cysts were found in horses and deer. In terms of cattle, only the non-zoonotic assemblage E of *G. intestinalis* has been found in 36 faecal samples from livestock using a real-time PCR assay [118]. Although the sample size is limited, the results of this study suggest a less important role of livestock in the transmission of *Giardia* to humans in Slovenia.

## Discussion

Analysis of the data obtained from a total of 10 countries showed that both *Cryptosporidium* spp. and *Giardia* spp. are commonly found in animals and in the environment when investigated, while giardiasis is more commonly reported in humans than cryptosporidiosis. Based on the number of reported cases in the ECDC Surveillance Atlas of Infectious Diseases, the difference between western Europe and eastern Europe appears more striking for cryptosporidiosis than for giardiasis [11].

Both parasites are prevalent in eastern Europe, but the number of reported cases varies greatly between the investigated countries; the causes of this variation include true differences in exposure and susceptibility, variable provision and access to healthcare systems, and differences in case definition, laboratory diagnosis, recording of cases and reporting. The national health systems of the countries covered here operate differently. Eight countries are members of the EU, and in these, both cryptosporidiosis and giardiasis are notifiable. In Bosnia and Herzegovina, neither disease is notifiable, and in Serbia, only giardiasis is notifiable. The different reporting standards may lead to varied levels of underreporting and varied recognition of the diseases as a public health issue. Making a disease mandatorily notifiable is an important step for obtaining accurate data, however, the quality and representativeness of the data obtained depends strongly on which patients are tested and which diagnostic tests are used. In many countries, neither the number of samples investigated nor the methods used for testing are reported. In our opinion, more transparency and uniformity in the collection of surveillance data are needed to further improve its quality. Currently, data available from the ECDC Surveillance Atlas of Infectious Diseases does not allow for reliable inter-country comparisons as demonstrated by the discrepancy in the reported occurrence of both diseases in humans when comparing surveillance data available via in the ECDC Surveillance Atlas of Infectious Diseases with the data provided by the public health laboratories (Table 1). Some countries provided lower or higher notification rates than that reported by public health laboratories. For example, no evidence of human infections of *G. intestinalis* was recorded for Romania in the ECDC Surveillance Atlas of Infectious Diseases (Table 1). Primary care doctors or physicians frequently treat patients with diarrhoeal disease symptomatically, without testing faecal samples for pathogens.

Another striking observation of our analysis is the discrepancy in the number of human cases between official reports of public health authorities (Table 1) and research-derived data. Although routine investigations and research studies are never directly comparable, the studies indicate more human infections than what is reflected in the routine investigations, therefore suggesting under-reporting throughout eastern Europe. One reason why research studies report more cases than public health authorities may be the ability to use more sophisticated methodology than that available for routine purposes. Under-reporting, which leads to underestimation of the burden of infection, is further anticipated because not all infected individuals exhibit clinical symptoms and some symptomatic persons do not seek medical care.

Data on the occurrence of *Cryptosporidium* spp. and *Giardia* spp. in animals in eastern Europe differ broadly in terms of targeted animal species and depth of analysis. This review showed that both *Cryptosporidium* spp. and *Giardia* spp. are common parasites of domestic animals, including pets, in eastern Europe, and importantly, genotypes pathogenic to humans, including *C. parvum* and *G. intestinalis* assemblage A and B*,* are prevalent. *C.parvum* subtype IIaA16G1R1 is a common subtype in the region, found in both cattle and humans in the Czech Republic, Estonia, Hungary, Romania and Slovenia [62,69,89,117,119]. It has also been suggested that birds may be carriers of human pathogenic species and genotypes of *Giardia* and *Cryptosporidium* [70].

Analysis of the current status of research on *Cryptosporidium* spp. and *Giardia* spp. in the environment highlighted that to date, relatively little is known about the occurrence and genetic diversity of these parasites in natural water supplies. Reports were available from the Czech Republic, Hungary, Poland and Romania [24,55-58,72,73,80-84,96-97].

Reports on presence of *Cryptosporidium* spp. and *Giardia* spp. in food were scarce from this region. Waterborne and food-borne outbreaks are clearly important to establish the burden of disease, but it is likely that many smaller outbreaks are currently missed [120,121].

Baseline data as well as improved understanding of the epidemiology, infection sources, reservoirs and transmission of cryptosporidiosis and giardiasis in eastern Europe are needed. Surveillance studies and outbreak investigations using molecular tools at the subtype level are warranted. In addition, consensus and updated methods that are harmonised across countries are required to make the data more comparable. Reducing public health risks from zoonoses and other threats at the human-animal-ecosystem interface must consider the complexity of interactions among humans, animals and the various environments in which they live. This requires communication and collaboration among the sectors responsible for human health, animal health and the environment in a One Health approach. Although the presented results may be important for public health specialists, epidemiologists, drinking and wastewater managers, veterinarians, farmers and the public in general, further addressing the knowledge gaps in a timely manner would greatly contribute to understanding the complex picture of cryptosporidiosis and giardiasis epidemiology and thus set the stage for appropriate future control plans.
